# The Role of Rare Copy Number Variants in the Functional Outcomes of Individuals With Neurodevelopmental Conditions

**DOI:** 10.1192/bjo.2024.142

**Published:** 2024-08-01

**Authors:** Maria Flanagan, Charlotte Dennison, Joanna Martin, Anita Thapar

**Affiliations:** Cardiff University, Cardiff, United Kingdom

## Abstract

**Aims:**

Copy number variants (CNVs) are large changes in the structure of DNA. Certain rare CNVs are associated with elevated chance of neurodevelopmental conditions and difficulties (NDs), including autism spectrum disorder (ASD) and intellectual disability, alongside various physical health complications. Currently, CNV testing in children with NDs is only recommended under limited circumstances, in part because their impact on outcomes and prognosis remains unknown. We aimed to investigate whether individuals with NDs in childhood, with and without rare pathogenic CNVs, differ in terms of functional outcomes in early adulthood.

**Methods:**

Pathogenic CNV carriers were identified in the Avon Longitudinal Study of Parents and Children (ALSPAC), a UK birth cohort of individuals born in 1991–1992. Individuals with the following childhood NDs were identified through parent-reported diagnostic interviews and questionnaires, and assessment with the child: Attention Deficit Hyperactivity-Disorder (ADHD), ASD, reading difficulties, coordination difficulties, language difficulties, and chronic tics. Outcomes were measured at age 25 and included: presence of an emotional disorder, being in receipt of sickness/disability benefit, ability to make and maintain friendships, not being in education, employment, or training (NEET), and self-reported life satisfaction. We will use logistic regression to measure the association between carrying a pathogenic CNV and each functional outcome in ALSPAC. Sensitivity analyses will be conducted on all large (>250kb), rare (<1%) CNVs, as opposed to only pathogenic CNVs.

**Results:**

983 individuals with probable NDs (39.4% female, n = 387) were identified in ALSPAC, including 495 people with ASD, 163 with ADHD, 16 with Tourette's syndrome, 210 with reading difficulties, 295 with language difficulties, and 166 with coordination difficulties. Many individuals met criteria for more than one ND.

43 (4.4%) of individuals with an ND carried a pathogenic CNV. CNV carrier status amongst individuals with a ND was not associated with sex (4.4% of females vs 4.4% of males, OR = 1.007 [0.539–1.882] p = 0.981). Analysis of CNV carrier status on outcomes in NDs will be conducted between February and April 2024.

**Conclusion:**

Evidence in support of poorer outcomes in CNV carriers could suggest that neurodiverse young people with CNVs may benefit from intervention to improve outcomes, and thus more individuals may benefit from genetic testing. Conversely, evidence indicating that CNVs do not impact outcomes may suggest that current clinical guidelines are appropriate within the current research landscape, and that further research is needed to understand the impact of carrying a pathogenic CNV in young people with NDs.

